# The Influence of Discrimination and Coping Style on Blood Pressure Among Black/African American Women in the InterGEN Study

**DOI:** 10.1089/heq.2019.0122

**Published:** 2020-06-29

**Authors:** Michelle L. Wright, Sungju Lim, Adam Sales, Shilpa Rajagopal, Dumebi Nzegwu, Cindy A. Crusto, Jacquelyn Y. Taylor

**Affiliations:** ^1^ School of Nursing, University of Texas at Austin, Austin, Texas, USA.; ^2^ Department of Women's Health, Dell Medical School, University of Texas at Austin, Austin, Texas, USA.; ^3^ SMARTER Consulting, College of Education, University of Texas at Austin, Austin, Texas, USA.; ^4^ Department of Biology, College of Natural Science, University of Texas at Austin, Austin, Texas, USA.; ^5^ Department of Health and Society, College of Liberal Arts, University of Texas at Austin, Austin, Texas, USA.; ^6^ Department of Psychiatry, Yale University School of Medicine, New Haven, Connecticut, USA.; ^7^ Department of Psychology, University of Pretoria, Pretoria, South Africa.; ^8^ School of Nursing, Columbia University, New York, New York, USA.

**Keywords:** African American, blood pressure, discrimination, coping style

## Abstract

**Purpose:** Although research has explored the effects of racism on mental health, few studies have investigated the effects of racism on physical health. In this study, we examined the influence of racial discrimination and race-related stress and coping on blood pressure within a cohort of Black/African American women.

**Methods:** This was a secondary data analysis of 226 Black/African American women from the Intergenerational Impact of Genetic and Psychological Factors on Blood Pressure study. Experiences of racial discrimination and coping, measured by the Experiences of Discrimination scale and the Race-Related Events Scale, were analyzed in relation to systolic blood pressure (SBP) and diastolic blood pressure (DBP). Multiple linear regression was used to explore the interaction effect of coping and discrimination on blood pressure for both scales.

**Results:** Age and elevated body mass index were associated with increased SBP and DBP, and low income was associated with increased DBP. Among individuals who reported no personal experience of discrimination, more active coping strategies were associated with higher DBP. There was no evidence of a relationship between type of coping strategies used and blood pressure among individuals who did report experiences of discrimination.

**Conclusion:** Differences in coping strategy in response to racism were not found to have a significant moderating effect on DBP in Black/African American women.

## Introduction

As the global community becomes smaller and health inequities become more visible within societies, there is an increasing interest in studying the influence of psychosocial factors on physiological health. External societal factors, including racism and socioeconomic status, are known to affect access to health care across the lifespan, and this can have detrimental long-term effects.^1^ Historically, medical research has stratified risk by racial/ethnic groups and gender. Hypertension and associated complications disproportionately affect Black/African American men and women.^2,3^ This disparity has persisted over time, and African American men and women continue to have twice the risk of hypertension than do their White counterparts.^4,5^ In the United States, African American women have the highest prevalence (46.1%) of hypertension among all racial/ethnic and gender groups.^6^ Hypertension is not only more severe among African Americans than among other racial groups, it also has an earlier onset.^7,8^ To date, however, the underlying cause for this persistent disparity in risk and prevalence of hypertension has not been fully elucidated.

Racial discrimination, job and economic insecurity, and social relationships have been identified as factors capable of increasing psychological stress that can contribute to the development of chronic conditions such as hypertension.^9^ African Americans and other racial/ethnic minorities are uniquely exposed to race-related stressors.^10,11^ Research exploring the interplay of racial discrimination and subsequent physiological effects in African Americans have identified positive correlations between both perceived discrimination^12^ and heightened race consciousness^13^ and frequency of chronic conditions such as high blood pressure.

Davis et al.^14^ found that the magnitude of African Americans' perceived stress associated with exposure to racial discrimination was a significant predictor for hypertension. Furthermore, workplace racism has been associated with increased systolic blood pressure (SBP) and diastolic blood pressure (DBP) levels in African Americans.^15^ Conversely, however, other studies with African Americans have not identified significant associations between increased experiences of racism itself and hypertension.^14,16^ Given these contradictory findings, it is possible that intrinsically driven factors such as coping strategies and the extent of internalization of stress-inducing experiences may modulate overall health outcomes.^17^

Sensitivity to social stressors may be influenced by the effectiveness of individual coping strategies. In a study of urban Black South African men, active coping approaches were associated with metabolic syndrome and increased risk for cardiovascular conditions.^18^ This suggests that when one experiences social and race-related stressors, active coping strategies may constitute an additional psychological burden associated with physiological health outcomes.^19^ Yet research on coping and SBP/DBP among African Americans has found varying results across coping styles, suggesting a need for greater evidence to link coping styles with cardiovascular health.^20^ Furthermore, few studies have examined how discrimination may influence physiological health specifically among African American women.

In this study, we examine the effects of race-related stress and experiences of discrimination and the mediating role of coping strategy style on blood pressure among African American women. The aims of this study were to (1) investigate the influence of racial discrimination on blood pressure among African American women in the Intergenerational Impact of Genetic and Psychological Factors on Blood Pressure (InterGEN) study,^21,22^ and (2) determine whether coping style mediates the effect of discrimination on blood pressure.

## Methods

### Study design and population

This is a secondary analysis of data from the InterGEN study.^21,22^ The goal of the parent InterGEN study was to examine the influence of genetic and psychological factors on blood pressure among African American mother–child dyads over time. A detailed description of the data collection methods and psychological measures used in these analyses has been previously reported.^22^ In brief, 250 mother–child dyads were recruited from preschool education centers and community events in Connecticut. To be included in the study, women had to be 21 years or older, self-identify as African American or Black, speak English, and have a biological child 3–5 years old and not have a psychiatric or cognitive disorder that may limit the accuracy of reporting. The study was approved by New York University's Institutional Review Board (approval no. 1311012986).

### Measures

#### Demographic data

Demographic data were collected at participants' baseline visit and at subsequent visits if subject to change (e.g., household income): age, level of education (three levels: less than high school, high school graduate, some college or higher), household income (three levels: <$15,000/year, 15,000–35,000/year, ≥$35,000/year), and health insurance (four levels: private, Medicaid, government/Affordable Care Act [ACA], none/other).

#### Health characteristics

Health characteristics were measured and collected at each study visit. Participants were asked about their current smoking status (yes/no), whether they had a previous diagnosis of hypertension (yes/no), and whether they were currently taking blood pressure medications (yes/no). Participants' body mass index (BMI) was calculated from their height and weight obtained at the time of the visit. Blood pressure was measured according to The Seventh Report of the Joint National Committee on Prevention, Detection, Evaluation, and Treatment of High Blood Pressure recommendations, and the average of three blood pressure readings obtained during the visit was used for SBP and DBP.^23^

#### Discrimination

Perceived discrimination was measured using two separate instruments: (1) the Race-Related Events Scale (RES)^24^ and (2) the Experiences of Discrimination (EOD) scale.^25^ The RES is a 23-item screening tool designed to be consistent with standard diagnostic definitions of traumatic events (*DSM-IV-TR* [Diagnostic and Statistical Manual of Mental Disorders, 4th edition, text revision]).^26^ Participants answered each item with “yes” (1) or “no” (0) to indicate if they have experienced different types of race-related events because of their race or ethnicity (e.g., “someone beat me or hurt me because of my race or ethnicity”). Total scores ranged from 0 to 22, with higher scores indicating greater perceived discrimination.

The EOD scale measured self-reported experiences of racial discrimination. We used the scale's 11-item version, which consists of two parts: two items that ask about coping with discrimination, and nine items that ask about situations in which a participant had experienced racial discrimination. For experienced discrimination, participants answered each question with “yes” or “no” (e.g., “Have you ever experienced discrimination, been prevented from doing something or been hassled or made to feel inferior in any of the following situations because of your race, ethnicity, or color?”). The nine situations are “at school,” “getting hired or getting a job,” “at work,” “getting housing,” “getting medical care,” “getting service in a store or restaurant,” “getting credit, bank loans, or a mortgage,” “on the street or in a public setting,” and “from the police or in the courts.” The EOD discrimination score was the sum of situations in which discrimination was experienced.

The EOD scale's two items for coping measure strategies employed by participants when they felt they were being treated unfairly.^25,27^ The first question asked whether participants “Accept it (discrimination) as a fact of life” (0) or “Try to do something about it” (1). The second question asked whether participants “Keep it to yourself” (0) or “Talk to other people about it” (1) when they have EOD. The total coping score was the sum of the two answers: engaged/active (2), moderate/neutral (1), or passive (0) coping. This score was treated as a continuous variable, as in the study by Krieger and Sidney^25^ We used this measure to evaluate coping because it is specific to coping with discrimination.

### Statistical analysis

The sample for data analysis comprised 226 women; 24 of the study's 250 original participants were excluded from analysis because of missing data. Data distributions were evaluated for normality. Scores for both discrimination measures (RES and EOD) were extremely right-skewed. We therefore dichotomized both measures as *no perceived experiences of discrimination* (total score=zero) and *experiences of discrimination* (score >0) and treated discrimination as a categorical variable. Descriptive statistics were used to evaluate socioeconomic status, hypertension-related health status and health behavior, experience of racial discrimination, and coping response toward racial discrimination.

Differences in blood pressure based on experience of discrimination and coping were evaluated with *t* tests or Pearson correlations. Multiple linear regression models were conducted separately for the effects of EOD and RES discrimination and coping on SBP and DBP. Continuous independent variables and covariates (i.e., coping, age, BMI) were centered to their sample mean values.^28^ Assumptions of linear regression such as linearity, homoscedasticity, and normality of residuals were assessed and met. Significance was established with a two-tailed alpha, *p*<0.05. Analyses were completed in RStudio version 1.1.456 (Boston, MA) with the following R packages: tidyverse,^29^ dplyr,^30^ and finalfit.^31^

## Results

The women in this study were on average 31.22 years old, with most <40 years (92.9%). A majority were overweight or obese (70.3%), and 23.5% identified as current smokers. More than half had finished some college or more (58.8%). Nearly half (47.8%) reported that their annual household income was <$15,000, and most participants used Medicaid or government/ACA insurance (78.3%) (Table 1). Forty-eight had previously been diagnosed with hypertension before enrolling in the study, and 8.4% were currently taking medication for hypertension. Nearly 25% had blood pressure readings that met diagnostic criteria for hypertension (≥130/80 mmHg).

**Table 1.** Sample Characteristics (*n*=226)

Characteristics	M±SD or *n* (%)
Age (years)	31.22±5.7
20s	95 (42.0)
30s	115 (50.9)
40s	16 (7.1)
BMI (kg/m^2^)	29.94±8.35
Underweight	13 (5.8)
Normal	54 (23.9)
Overweight	57 (25.2)
Obese	102 (45.1)
Current smoking status	
Yes	53 (23.5)
Education	
<High school	12 (5.3)
High school graduate	81 (35.8)
Some college/graduate	133 (58.8)
Annual household income	
<$15,000	108 (47.8)
$15,000–34,999	68 (30.1)
>$35,000	50 (22.1)
Health insurance	
Private	32 (14.2)
Medicaid	141 (62.4)
Government/ACA	36 (15.9)
None or others	17 (7.5)
Blood pressure^a^	
Systolic	115.31±15.96
Diastolic	73.27±11.27
Categorized into HTN	54 (23.9)
Diagnosed HTN^b^	
Yes	48 (21.2)
Current HTN medication	
Yes	19 (8.4)

^a^ Adjusted blood pressure: if taking HTN medication, add 15 mmHg to SBP and 10 mmHg to DBP.

^b^ HTN=dichotomized with SBP ≥130 mmHg or DBP ≥80 mmHg.

ACA, Affordable Care Act; BMI, body mass index; DBP, diastolic blood pressure; HTN, hypertension; *M*, mean; SBP, systolic blood pressure; SD, standard deviation.

More than half of the sample indicated that they had experienced discrimination based on their race (51.8% and 58.0% for the EOD and RES, respectively). On average, participants were more likely to have used active coping strategies when experiencing racial discrimination; higher scores were associated with active versus passive coping (Table 2). Within this cohort, we did not observe significant associations between SBP or DBP and either measure of racial discrimination (EOD or RES). However, we did identify a significant positive relationship between coping and DBP (Pearson's *r*=0.17, *p*=0.012; Table 2).

**Table 2.** Discrimination, Coping, and Unadjusted Association with Blood Pressure (*n*=226)

Characteristics	M ± SD or *n* (%)	SBP (mmHg)		DBP (mmHg)	
		M ± SD	*t* or *r* (*p*)	M ± SD	*t* or *r* (*p*)
Racial discrimination					
EOD					
Never experienced	109 (48.2)	115.54 ± 15.49	0.21 (0.831)	73.41 ± 11.95	0.19 (0.851)
Experienced	117 (51.8)	115.09 ± 16.45		73.13 ± 10.65	
RES					
Never experienced	95 (42.0)	116.96 ± 15.28	1.33 (0.185)	74.43 ± 11.41	1.32 (0.188)
Experienced	131 (58.0)	114.11 ± 16.39		72.43 ± 11.13	
Coping: response to unfair treatment	1.59 ± 0.60		0.11 (0.101)		0.17 (0.012)^*^

^*^ *p*<0.05.

EOD, Experiences of Discrimination; RES, Race-Related Events Scale.

When we examined the predictive utility of discrimination and coping for blood pressure, adjusting for confounding factors (i.e., age, BMI, smoking status, education, and income), neither discrimination nor coping contributed significantly to SBP more than age or BMI (Table 3). Models with and without risk associated with discrimination and coping strategies explained 26% of the variance in blood pressure, with age and BMI positively associated with SBP. In models evaluating the association of discrimination, coping strategy, and DBP, only age and BMI were both predictive of DBP. An increase of 1 year of age corresponded to an increase of 0.63 mmHg DBP (95% confidence interval [CI]=0.39–0.88), and a 1 U increase of BMI corresponded to an increase of 0.49 mmHg of DBP (95% CI=0.33–0.65). Coefficient estimates were similar in models 2 and 3. Adding discrimination and coping strategy into the model accounted for only an additional 1% of variance in DBP.

**Table 3.** Adjusted Association Between Blood Pressure and Racial Discrimination, Coping, and Demographic Variables (*n*=226)

		EOD model		RES model	
	Step 1	Step 2	Step 3	Step 2	Step 3
	*B*	*B*	*B*	*B*	*B*
SBP					
Age	0.80^***^	0.77^***^	0.78^***^	0.77^***^	0.78^***^
BMI	0.76^***^	0.76^***^	0.76^***^	0.75^***^	0.75^***^
Smoking					
Yes (vs. no)	3.35	3.34	3.40	3.22	3.21
Education					
High school (vs. <high school)	−5.04	−4.99	−5.13	−4.81	−4.71
Some college/graduate (vs. <high school)	−5.18	−4.91	−5.04	−4.45	−4.35
Income					
$15,000–34,999 (vs. <$15,000)	−2.81	−3.14	−3.08	−2.68	−2.77
>$35,000 (vs. <$15,000)	−0.97	−1.34	−1.33	−1.08	−1.13
Discrimination					
Yes (vs. no)		−0.49	−0.51	−2.32	−2.30
Coping		1.77	2.62	1.92	1.49
Discrimination^*^coping			−1.75		0.82
*F* (*p*)	12.32 (<0.001)	9.70 (<0.001)	8.73 (<0.001)	9.91 (<0.001)	8.89 (<0.001)
Adjusted *R*^2^	0.26	0.26	0.26	0.26	0.26
DBP					
Age	0.63^***^	0.59^***^	0.61^***^	0.60^***^	0.60^***^
BMI	0.49^***^	0.49^***^	0.49^***^	0.48^***^	0.48^***^
Smoking					
Yes (vs. no)	0.83	0.80	0.92	0.71	0.71
Education					
High school (vs. <high school)	−0.99	−0.92	−1.23	−0.79	−0.81
Some college/graduate (vs. <high school)	−1.63	−1.32	−1.61	−0.97	−0.99
Income					
$15,000–34,999 (vs. <$15,000)	−3.33^*^	−3.81^*^	−3.67^*^	−3.46^*^	−3.45^*^
>$35,000 (vs. <$15,000)	−1.47	−1.99	−1.96	−1.79	−1.78
Discrimination					
Yes (vs. no)		−0.24	−0.28	−1.70	−1.70
Coping		2.42^*^	4.22^**^	2.53^*^	2.61
Discrimination^*^coping			−3.73		−0.15
*F* (*p*)	11.31 (<0.001)	9.43 (<0.001)	8.85 (<0.001)	9.65 (<0.001)	8.65 (<0.001)
adj. *R*^2^	0.24	0.25	0.26	0.26	0.25

^*^ *p*<0.05; ^**^p<0.01; ^***^p<0.001.

Individuals who adopted an active coping strategy tended to have a higher average DBP than did those who used passive or neutral coping styles. The relationship between active coping and higher DBP was evident only in individuals who had not reported EOD, and not in individuals who had reported such experiences. Although our models using the RES to evaluate EOD approximated those using the EOD measure, no significant interaction effect was observed with the RES scale (Table 3).

## Discussion

The results of this study indicate that age and BMI were strong predictors of high blood pressure among participants, which is consistent with previous evidence.^2^ Furthermore, additional evidence suggests that BMI influences blood pressure more strongly among women than among men, even among premenopausal women represented in our cohort.^32^ Although our participants were relatively young (i.e., 31 years), >20% were diagnosed with hypertension before enrolling in the study and >70% were overweight or obese. Elevated BMI may have contributed to the high prevalence of early high blood pressure among our participants.

The present results are consistent with those of other studies in which being of reproductive age has conferred protection from cardiovascular disease only in lean women of reproductive age, relative to their male counterparts.^33^ A review by Faulkner and Belin de Chantemèleet al. suggested physiologic mechanisms that contributed to high blood pressure among premenopausal women related to obesity are not because of sympathetic nervous system activation, as observed among men.^33^ This may be explained by the difference in distributions of adipose tissue accumulation among women of reproductive age, which is primarily subcutaneous versus visceral more common among men and postmenopausal women. The subcutaneous fat releases leptin, which may activate the aldosterone–mineralocorticoid receptor axis and contribute to the development of hypertension. If the development of hypertension among premenopausal women with high BMI is strongly driven by obesity-induced mechanisms, it may be difficult to detect hypertension-inducing effects of other factors that influence blood pressure.

Nearly half of the women participating in our study reported that they had not experienced discrimination based on their race or skin color. Although several factors may influence perception of perceived racial discrimination, other studies have demonstrated lower levels of perceived discrimination and depression among Black men living in predominantly Black neighborhoods as opposed to predominately White neighborhoods.^34^ Many of the women participating in our study lived in predominantly African American neighborhoods, which may have decreased the frequency of experiences of interpersonal racism. Another study has reported that experiences of discrimination among African Americans were impacted by colorism, such that women with lighter skin tones reported fewer experiences of discrimination.^35^ Our study did not collect skin tone data, and future consideration of skin tone might be useful in evaluating perceived racism related to health outcomes.

As in previous studies, we did not identify a significant association between increased experiences of racism and blood pressure.^14,16^ Instead, we did find that coping style in situations not associated with discrimination were associated with elevated DBP. In general, when coping with racism more actively, women showed higher DBP than did those who adopted passive or neutral coping styles. Of interest, this is comparable with what has been observed among Black men, who have increased cardiovascular risk when employing active coping.^18^ However, a recent study by Barajas et al. determined that active coping was associated with decreased SBP among men, but not among women, after adjusting for experiences of discrimination.^36^ Unfortunately, we cannot compare our results related to DBP among women with the Barajas study, because DBP was not analyzed or reported in their study.

Historically, race, not racism, has been included as a risk factor or covariate in analyses related to physical health outcomes. A substantial body of literature has evaluated experiences of racial discrimination in relation to stress and mental health outcomes, with less research evaluating direct and indirect influences on physiological health.^37^ Others have observed differences in mental health outcomes when assessing individual coping style in relation to EOD. Thomas et al.^38^ determined that cognitive-emotional coping styles partially mediated the stress response among African American women to gendered racism. To our knowledge, this study is the first to take the next step by evaluating the influence of experiences of racial discrimination and coping style on blood pressure among African American women.

### Health equity implications

With increasing research on precision health care, it remains important to contextualize individual health risks. Health research frequently focuses on identifying risk factors that are controllable at the individual level; yet, the influence of racism on physical and mental health suggests that individual intervention strategies may be ineffective in mitigating risk.^39^ In this study, however, DBP outcomes varied with coping strategies. Accordingly, future research might examine longitudinal data and experiences to identify whether certain coping mechanisms are more protective with regard to mitigating health risks.

Nevertheless, focusing on developing individual coping strategies to reduce health risk related to racism will not eliminate the problem of racism itself or actual risk. To address such health inequities, we must focus on dismantling structural racism instead of placing an onus on those experiencing racism to cope with persistent structural inequities.^40^ To accomplish this, Hardeman et al.^40^ have recommended several strategies, including centering our work among the communities we serve. For example, given that health research frequently uses *white* as its reference (i.e., “normal”) group, we must actively redefine “normal” and focus on incorporating sustainable anti-racist strategies, instead of merely being inclusive.

## Conclusion

Previous studies have reported disparate associations between experiences of racism and blood pressure among African Americans. In our cohort, active coping strategies were associated with higher DBP among Black/African American women who had not experienced racial discrimination, and there was no significant relationship with coping strategy and blood pressure among those experiencing discrimination. Future research is needed to investigate the longitudinal impact of race-related discrimination and coping strategies on associated physiological health outcomes.

## Abbreviations Used

ACA: Affordable Care Act
BMI: body mass index
CI: confidence interval
DBP: diastolic blood pressure
EOD: Experiences of Discrimination
HTN: hypertension
InterGEN: Intergenerational Blood Pressure
*M*: mean
RES: Race-Related Events Scale
SBP: systolic blood pressure
SD: standard deviation
